# Emerging Role of Mitophagy in the Heart: Therapeutic Potentials to Modulate Mitophagy in Cardiac Diseases

**DOI:** 10.1155/2021/3259963

**Published:** 2021-09-23

**Authors:** Yi Luan, Ying Luan, Qi Feng, Xing Chen, Kai-Di Ren, Yang Yang

**Affiliations:** ^1^Department of Translational Medicine Center, The First Affiliated Hospital of Zhengzhou University, Zhengzhou 450052, China; ^2^Department of Physiology and Neurobiology, School of Basic Medical Sciences, Zhengzhou University, Zhengzhou 450001, China; ^3^Research Institute of Nephrology, Zhengzhou University, Zhengzhou 450052, China; ^4^Department of Pharmacy, The First Affiliated Hospital of Zhengzhou University, Zhengzhou 450052, China; ^5^Henan Key Laboratory of Precision Clinical Pharmacy, Zhengzhou University, Zhengzhou 450052, China

## Abstract

The normal function of the mitochondria is crucial for most tissues especially for those that demand a high energy supply. Emerging evidence has pointed out that healthy mitochondrial function is closely associated with normal heart function. When these processes fail to repair the damaged mitochondria, cells initiate a removal process referred to as mitophagy to clear away defective mitochondria. In cardiomyocytes, mitophagy is closely associated with metabolic activity, cell differentiation, apoptosis, and other physiological processes involved in major phenotypic alterations. Mitophagy alterations may contribute to detrimental or beneficial effects in a multitude of cardiac diseases, indicating potential clinical insights after a close understanding of the mechanisms. Here, we discuss the current opinions of mitophagy in the progression of cardiac diseases, such as ischemic heart disease, diabetic cardiomyopathy, cardiac hypertrophy, heart failure, and arrhythmia, and focus on the key molecules and related pathways involved in the regulation of mitophagy. We also discuss recently reported approaches targeting mitophagy in the therapy of cardiac diseases.

## 1. Introduction

**Figure 1:**
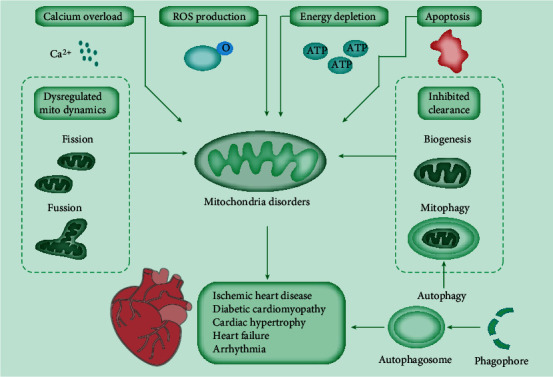
Mitochondrial dysfunctions involved in cardiac disease. Cardiac mitochondria play an important role in energy supply, calcium storage, cell apoptosis, and ROS production. Mitochondrial dynamics through fission and fusion cycles, biogenesis, and mitophagy modulate the mitochondrial pool. Many factors can induce mitochondrial disorders, such as calcium overload, dysregulated mitochondrial dynamics, energy depletion, increased oxidative stress, inhibited damaged mitochondria clearance, and cell apoptosis. Efficient autophagy is essential for the homeostasis of cardiac metabolism. The defects of autophagy or its special type, mitophagy, would ultimately lead to heart-related diseases. The consequences of mitochondrial dysfunction in the heart include ischemia/reperfusion injury, diabetic cardiomyopathy, cardiac hypertrophy, heart failure, and arrhythmias.

Mitochondrial metabolism is important to supply a tremendous energy demand for the heart [1]. Cardiac mitochondria are responsible for the generation of about 6–7 kg ATP each day by oxidative phosphorylation and take up 30% volume of cardiomyocytes [2]. Cardiac mitochondria have a critical role in energy supply; contribute to calcium storage, cell apoptosis modulation, and the necrosis pathway; and serve as a metabolic hub for the Krebs cycle and fatty acid *β*-oxidation [3]. Thus, the normal function of the mitochondria is critical for cardiac homeostasis. Aberrant mitochondrial function results in inadequate energy, thereby augmenting the generation of reactive oxygen species (ROS, Figure 1) [4]. ROS accumulation leads to damage to mitochondrial DNA, lipids, and proteins involved in respiration, thereby contributing to severe oxidative damage and cell death [5]. Such damage in different terminal tissues, such as the brain and heart, is featured by different diseases, as manifested by neurodegenerative and cardiovascular diseases [6].

Several compensating mechanisms function to maintain the proper homeostasis of mitochondrial function [7]. For example, the correct folding of mitochondrial proteins in extreme oxidative conditions is maintained by a series of mitochondrial chaperons and proteases [8]. If the capacity of these proteins is counteracted, cells utilize the unfolded protein response to compensate for the expression of chaperones and proteases and restore protein homeostasis [9]. Other mechanisms include alterations in mitochondrial morphology. For instance, mitochondrial fusion and fission allow for the exchange of matrix enzymes and mitochondrial DNA of different mitochondria to maintain a healthy network of interactions, and this phenomenon is commonly defined as mitochondrial dynamics [10]. Mitochondrial fusion is composed of the fusion of outer and inner mitochondrial membranes of different mitochondria [11]. The mitochondrial fusion is induced by specific proteins, including mitofusins (MFN) 1 and 2 on the outer membrane, and optic atrophy 1 on the inner mitochondrial membrane and intermembrane space [12]. Mitochondrial fission mediates the redistribution of mitochondria in cardiomyocytes and needs the interaction of a cytosolic protein, i.e., dynamin-related protein 1 (DRP1), and an outer membrane protein, i.e., MFN1 protein, thereby composing the mitochondrial fission complex [13]. Mitochondrial dynamics is the consequence of the consecutive balance of fusion and fission processes [14]. For instance, the disruption of mitochondrial fusion resolves mitochondrial fragmentation and induces apoptosis [15]. The suggested roles of fusion and fission have been assumed in many mitochondrial processes, such as mitochondrial DNA deletion and bioenergetics, and in different cardiac diseases, including cardiomyopathies and heart failure (HF) [16] (Figure 1).

Mitochondrial biogenesis contributes to the consecutive production of new mitochondria into networks [17]. In cardiomyocytes, the proper expansion of cardiac mitochondria is essential to maintain adequate energy supply for cardiac contractility. The mitochondrial biogenesis needs the incorporation of the transcriptional cascade, including the activation of PPAR coactivator 1, followed by increased nuclear respiratory factors, which are responsible for the expression of mitochondrial DNA and proteins [18].

Autophagy is involved in the maintenance of intracellular homeostasis in most types of cardiovascular cells, particularly cardiomyocytes. Mitophagy is an autophagic response that specifically targets damaged and, hence, potentially cytotoxic mitochondria. Therefore, genetic defects in autophagy or mitophagy have been identified to exacerbate the disposition to spontaneously develop cardiodegenerative disorders. Mice with cardiomyocyte-specific deletion of Atg5 developed cardiac hypertrophy, left ventricular dilatation, contractile dysfunction, and premature death accompanied by disorganized sarcomere structure, mitochondrial misalignment, and aggregation [19]. Also, the depletion of Pink1, a key mediator of mitophagy, caused left ventricular dysfunction and pathological cardiac hypertrophy in mice by 2 months of age [20] (Figure 1).

The interaction of these processes permits high adaptation to change metabolic environments. Once all of these mechanisms fail to restore the normal function of damaged mitochondria, cells initiate a removal process known as mitophagy, which selectively promotes the autophagosome to remove damaged organelles for the fitness of the mitochondrial network [21] (Figure 1).

Mitophagy, as a selective autophagy in mitochondrial damage, is first identified in yeast [22]. The common feature of mitophagy is the appearance of autophagic vacuole containing damaged mitochondria, which is also defined as a mitophagosome [23]. Mitophagy is crucial in cardiac myocytes. The mitophagy in the heart can be induced by hypoxia and superabundant generation of ROS (such as reperfusion) [24]. Aberrant mitophagy induces the accumulation of damaged mitochondria, reduction of myocytes, and dysfunction of contraction. The abnormalities in mitochondrial dynamics and mitophagy are directly related to the defective clearance of damaged mitochondria and inflammatory response activation, which ultimately contribute to cardiac aging and HF [25].

**Figure 2:**
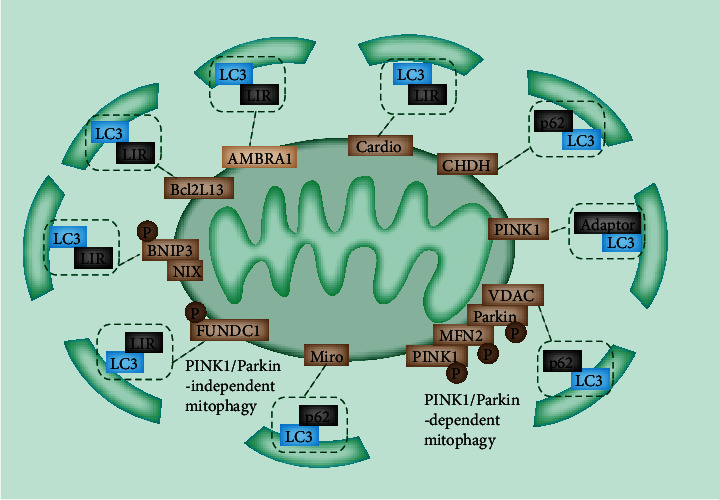
Major mitophagy pathways. PINK1/Parkin-mediated mitophagy. Upon mitochondrial depolarization under cell stress, PINK1 can no longer be imported into the mitochondria. The autophosphorylated PINK1 accumulates on OMM and recruits Parkin to the mitochondria. The E3 ligase Parkin polyubiquitylates OMM proteins VDAC1, MFN1, MFN2, and Miro1, which are recognized by LC3 adaptors on phagophore. *FUNDC1-mediated mitophagy*: under hypoxic conditions, PGAM5 dephosphorylates FUNDC1, restores FUNDC1 ability to interact with LC3 through LIR, and activates mitophagy. *BNIP3/NIX-mediated mitophagy*: BNIP3 and BNIP3L/NIX act synergistically and share an overlapping biological function in activating mitophagy when mitochondria have stable membrane potential. CHDH accumulates on the OMM and interacts with p62 and binds with LC3. AMBRA1, Bcl2L13, and cardiophospholipids directly recognize LC3 through LIR.

Mitophagy can be divided into three types: mitophagy induced by nutrient limitation, mitophagy induced by damage signals, and micromitophagy associated with small mitochondrion-derived vesicles [23]. These types are basically different because the first two types require the fusion to the lysosome to form an autophagosome surrounding the mitochondria, whereas the latter does not. As shown in Figure 2, mitophagy is closely supervised by a number of cellular signal mechanisms, including PTEN-induced putative kinase 1 (PINK1), Parkin, mitophagy receptors, and certain mitophagy adaptors [26]. Mitophagy receptors are principally localized on the outer mitochondrial membrane (OMM) via transmembrane domains and attach autophagosomes to the mitochondria through the LC3-interacting region (LIR) motif, including ATG32 in yeast, B-cell CLL/lymphoma 2- (BCL2-) interacting protein 3 (BNIP3), BNIP3-like (BNIP3L)/NIX, FUN14 domain-containing 1 (FUNDC1), Bcl2-like 13 (BCL2L13), and FKBP prolyl isomerase 8, in mammalian cells [27]. These mitophagy receptors recruit ATG8 family protein LC3 and its homolog gamma-aminobutyric acid receptor-associated protein to the mitochondrial membrane and induce the initiation of mitophagy independent of the ubiquitin pathway with the aid of the LIR motif [27].

## 2. PINK1

Mitophagy can be dependent or independent to phosphatidylinositol-3,4,5-trisphosphate 3-phosphatase PINK1 [23]. Thus far, PINK1/Parkin-dependent mitophagy remains the most extensively characterized mitophagy (Figure 2). PINK1, a serine/threonine kinase, remains at a low level in normal cells [28]. Upon mitochondrial damage, PINK1 accumulates at the OMM, triggering elevated mitochondrial ROS, depolarization, and increased misfolded proteins. Ascending PINK1 autophosphorylates, activates itself, and phosphorylates ubiquitin, thereby translocating Parkin to the mitochondrial membrane [29]. As an E3 ligase, the Parkin translocation and activation lead to the ubiquitination of mitochondrial proteins and autophagy [30]. The recently described protein ubiquitin carboxyl-terminal hydrolase 30 exerts an inhibitory effect on the Parkin-mediated mitophagy [29]. Interestingly, Parkin can also induce mitophagy in the absence of PINK1 by promoting mitochondrial depolarization [31]. The ligase activity of Parkin can be modulated by some identified proteins at OMM, including MFN1/2, voltage-dependent anion channel protein 1 (VDAC1), and mitochondrial Rho guanosine triphosphate hydrolases (MIRO) [23]. Therefore, the dynamics in OMM are important for mitophagy. PINK1 is recruited into the mitochondria and cleaved by the mitochondrial processing peptidase to maintain a remarkably low level of PINK1 in healthy cells [32]. After cleavage, PINK1 is ubiquitinated and degraded by the ubiquitin proteasome system.

Several pathways and proteins are involved in PINK1-dependent mitophagy. MFN2 can be phosphorylated by PINK1 and act as a Parkin receptor to remove damaged mitochondria [33]. VDAC1, a key protein in mitochondria-dependent apoptosis, is identified to be ubiquitinated through the interaction with Parkin and is involved in Parkin recruitment [34]. By contrast, defects in ubiquitination contribute to apoptosis and suppress mitophagy [35]. Therefore, the interaction between VDAC1 and PINK1 acts as a determinant in terms of damaged mitochondrial apoptosis and mitophagy [36]. In addition, MIRO1/2 can be ubiquitinated by Parkin [37]. MIRO1/2 are components of the adaptor complex, which recruits the mitochondria to motor proteins [38]. Thus, MIRO1/2 are involved in Ca^2+^-induced axonal mitochondrial movement. Upon calcium binding, the adaptor complex dissociates from microtubules and inhibits mitochondrial movement, further promoting mitophagy to eliminate damaged mitochondria [38].

## 3. Light Chain 3 (LC3)

Ubiquitination is an important process in selective autophagy for all cases [39]. Cargo-bound receptors recruit the microtubule-associated protein 1 LC3 (MAP1LC3) by LIR remains a well-established model, linking cargo with preformed, autophagy-generated membrane [40] (Figure 2). In this model, receptors are either a component of cargo or attracted to the cargo by ubiquitination. Additionally, scaffold proteins are essential for the recruitment of additional autophagy-related proteins [41]. In mitochondria, ubiquitin-bound adaptor proteins are responsible for the transport of depolarized mitochondria to the perinuclear region via the microtubule after OMM remodeling induced by the degradation of ubiquitinated proteins [41]. The interaction of adaptor proteins and LC3 facilitates the sequestration of damaged mitochondria into autophagosomes [42]. Subsequently, the fusion of autophagosomes with lysosomes promotes the degradation of damaged mitochondria.

Cargo-binding receptors (LC3 adaptors) can be recognized by LC3 and recruited to polyubiquitinated substrates on the mitochondria through their ubiquitin-binding domain, including sequestosome-1 (p62), nuclear domain 10 protein 52, optineurin, Trans-activating transcriptional regulatory protein of HTLV-1-binding protein 1, and neighbor of breast cancer 1 gene 1 [43].

## 4. Ubiquitin-Independent Mitophagy

A previous study indicated that autophagy and mitophagy are activated in PINK1-deficient cells [44]. Damaged mitochondria can also be eliminated independently of ubiquitination by the LC3 adaptor. These adaptors perceive damaged mitochondria directly and subsequently alter their subcellular localization or their interacting proteins, thereby translocating damaged mitochondria to autophagosomes. The best example implicated in mitochondrial removal is the BCL2/adenovirus E1B 19 kDa-interacting protein 3 (BNIP3) and BCL2/adenovirus E1B 19 kDa-interacting protein 3-like (NIX/BNIP3L) pathways [45]. BNIP3 and NIX are reported to induce mitophagy in response to hypoxia and induce mitochondrial depolarization and fusion with cell membranes [45]. The N-terminals of BNIP3 and NIX in the cytoplasm induce mitophagy through the interaction with LC3-related molecules. The direct binding of BNIP3 stabilizes itself and promotes the recruitment of Parkin [46].

## 5. Additional Parkin-Independent Mitophagy

Many other Parkin-independent mechanisms, including receptor-mediated mechanisms, exist. For example, FUNDC1, an OMM-localized protein, can initiate mitophagy by recruiting the MAP1LC3B/LC3B through its LIR motif in mammalian cells [47]. The FUNDC1 activity is modulated by phosphorylation via several different kinases [48]. The dephosphorylation mediated by PGAM5 is required to activate and interact with LC3, thus leading to mitophagy [49]. Choline dehydrogenase (CHDH) accumulates in the OMM in response to damaged mitochondrial potential, which is normally localized in IMM and OMM [50]. CHDH associates with p62 and forms the CHDH-p62-LC3 complex that mediates mitophagy. Notably, the monophosphate-activated protein kinase (AMPK), a nutrient deprivation sensor, can also induce Parkin-independent mitophagy through the TBK1 phosphorylation and activation [51].

## 6. Novel Regulatory Pathways

Recent publications also mentioned the physiologically related LC3-independent mitophagy. The Rab9-mediated autophagosome drives mitophagy by forming Rab9, Unc-51-like kinase 1 (ULK1), and DRP1 protein complex [52]. Furthermore, accelerating novel pathways involved in mitophagy continue to be identified. For instance, SMAD-specific E3 may modulate autophagy [53]. The autophagy protein coiled-coil myosin like BCL2-interacting protein (BECN1)/Beclin1 exerts a critical role in autophagosome formation and maturation and interacts with Parkin but is independent of its translocation to mitochondria [54]. At present, accumulating studies have associated mitophagy with ER stress via the specialized ER-mitochondrial contact regions (MAMs) that are responsible for Ca^2+^ fluxes and apoptosis modulation [55, 56]. The critical function of MAMs in mitophagy is well established because they are indispensable for the initiation autophagy, and many autophagy-related proteins are located in MAMs [56]. PINK1 and Beclin1 are identified to relocalize in MAMs to facilitate the contact of mitochondria with ER and the formation of the autophagosome in response to mitophagy stimulation [54].

## 7. Mitophagy and Cardiac Disease

Considering the critical role of mitophagy in the removal of damaged mitochondria, accumulating evidence believes that alterations in mitophagy can contribute to the progression of cardiac disease [57]. Notably, PINK1 is highly expressed especially in the heart. PINK1 is downregulated in the heart tissues of patients with late-stage HF compared with those of normal controls [58]. Moreover, the ablation of PINK1 in mice induces cardiac hypertrophy at two months of age, and PINK1 knockout aggravates the infarct size after ischemia/reperfusion (I/R) injury (Table 1).

### 7.1. Ischemic Heart Disease

Myocardial damage in ischemic heart disease is the outcome of inadequate myocardial requirement and coronary blood flow, acutely threatening human health. Ischemia leads to cell damage and death in the myocardium. Therapies targeting coronary circulation can potentially restore cardiac injury. However, reperfusion causes irreversible cardiac damage, which is known as I/R injury [59]. The critical role of mitochondria in cardiac function makes mitochondria the main target of I/R injury. I/R induces damage to mitochondrial cristae, abnormality in mitochondrial membrane potential, and mitochondrial permeability transition pore (mPTP) and subsequently results in aggravated imbalance of mitophagy [60]. Mitophagy protects cardiac cells from I/R injury [61]. I/R injury inhibits mitophagy and induces apoptosis in myocardial cells. Despite these, mitophagy may cause adverse effects on I/R injury. Mitophagy inhibition can possibly alleviate the I/R injury of cardiomyocytes, decrease apoptosis, and improve cardiac function [62] (Table 1). PINK1 and Parkin are enhanced, and the Parkin translocation and activation are increased in I/R injury. Parkin ablation in young mice, which are sensitive to I/R injury, exhibits normal heart function [63]. Besides, during I/R injury, BNIP3 is upregulated, and FUNDC1 is downregulated [63]. The effect of I/R injury on mitophagy depends on the phosphorylation state of BNIP3. The increased phosphorylation at S17 in I/R injury can strengthen the binding of BNIP3 and LC3, thus promoting mitophagy [64]. I/R injury induces phosphorylation at Y18 and S13, which reduces LC3 binding and mitophagy [62]. Other mitophagy regulators also influence the process of I/R injury. For instance, Pgam5, a serine/threonine protein phosphatase, is also involved in I/R injury [65]. The depletion of Pgam5 in mice shows augmented infarct volume and is well correlated with mitophagy inhibition.

### 7.2. Diabetic Cardiomyopathy (DCM)

As a major phenotype of tissue damage caused by diabetes, DCM acts as the main source of morbidity and mortality in patients with diabetes [66]. Considering that mitochondria are the major site for ROS production and oxidative stress injury, the dysfunction in the mitochondria is an outstanding inducer of DCM [67]. In DCM, a huge amount of ROS is produced, further aggravating the injury in mitochondria and the invalid cycle of cardiomyocyte death [67]. Animal studies demonstrated effective antioxidative therapy in DCM. However, the continued production of ROS from damaged mitochondria potentially prevents the use of antioxidative therapy clinically [68]. The potential therapeutic target of DCM is the inhibition of the sustained production of ROS by mitophagy. The exact role and relevant regulatory mechanism of mitophagy in DCM remain elusive. In DCM, the mitophagy level is apparently different. The mitophagy status is suppressed in type 1 diabetes but enhanced in type 2 diabetes models [69]. The protection or destruction effect of mitophagy in DCM requires additional evidence. Mitochondrial ROS production is elevated, and the BNIP3 level in prediabetic rats is inhibited compared with that in control rats [70] (Table 1). PINK1 and Parkin levels are suppressed in type 1 and type 2 diabetes models. Mice subjected to Parkin ablation display enhanced mitophagy, serious cardiac hypertrophy, and diastolic dysfunctions under high-fat diet stress [71]. A recent study revealed that the inhibition of mitophagy results in the accumulation of heart lipid on high-fat diet [72]. Increased mitophagy by trans-activator of transcription-Beclin1 contributes to the suppression of cardiomyopathy development [73]. Melatonin therapy relieves DCM-induced mitochondrial function by boosting PINK1/Parkin-mediated mitophagy [74].

### 7.3. Cardiac Hypertrophy

As an adaptive compensatory mechanism, cardiac hypertrophy is recognized as “physiological” in normal cardiac functions and “pathological” in cardiac dysfunctions [75]. Growing evidence has shown different signaling pathways in physiological and pathological hypertrophy. Pathological hypertrophy is accompanied by reduced OXPHOS [76] and can be induced by multiple stimuli, including hemodynamic pressure, ischemia, and myocardial injury. Pathological hypertrophy can be beneficial at first because of the compensation in contractibility and myocardial function and induces irreversible structure remodeling, amplified secretion of inflammatory cytokine, cell dysfunction, and cell death. These phenomena ultimately promote the progression of heart disease and HF [77].

Cardiac remodeling is commonly accompanied by augmented autophagy, whose function in disease pathology remains unclear [78]. Some research believed in the detrimental effect of mitophagy activation in myocardium remodeling and fibrosis. Conversely, the impaired mitophagy induced by the loss of the key element of mitophagy can contribute to pathological cardiac remodeling. Parkin-depleted mice exhibit elevated cardiac remodeling, and PINK1-ablated mice show abnormal mitochondrial function, increased oxidative stress, and pathological hypertrophy [79] (Table 1). The depletion of BNIP3 also exhibits cardiac hypertrophy at old age [80]. Therefore, the activation and inhibition of mitophagy bring about an adverse outcome in the progression of cardiac hypertrophy and remodeling. Efforts are needed for the comprehensive understanding of mitophagy in this pathology.

### 7.4. Heart Failure

As the late stage of various kinds of heart disease, HF is featured by high morbidity and mortality. Present therapies of HF may relieve symptoms, but the prognosis remains unsatisfied. For the effective screening of the HF strategy, an improved understanding of oxidative stress and relevant chronic inflammation is necessary. HF is characterized by mitochondrial dysfunction. Along with aging or disease, mitophagy is receding, leading to inadequate removal of damaged mitochondria and elevated ROS and peroxide levels [81]. The accumulated oxidative damage of mitochondrial proteins, lipids, and DNA ultimately results in HF. Studies revealed reduced autophagy-specific genes *Beclin1* and *LC3-II* in the specimen of patients with HF (Table 1). Thus, mitophagy is assumed to be also correlated with the progression of HF. MFN2 is reported to modulate HF-associated mitophagy by changing the mitochondrial membrane potential [82]. Follow-up studies noticed that inadequate mitophagy further exacerbates heart injury. The PINK1 downregulation is commonly observed in patients with HF, suggesting reduced mitophagy level in HF [3]. However, the cause and effect of mitophagy and HF remain to be determined. PINK1-depleted mice are liable to heart stress and HF induced by overload. The Parkin-associated mitophagy weakens with age. The damaged mitochondrial number increases with age. When the balance of Parkin-mediated mitophagy and damaged mitochondria is broken, the mitophagy in cardiomyocytes is unable to maintain the normal function of mitochondria [83]. The Parkin depletion in mice induces accumulated abnormal mitochondria, which ultimately increases the risk of HF. The NIX depletion shows decreased myocardial fibrosis and normal systolic function in stress-induced HF mouse models [84]. No study has mentioned increased mitophagy with the occurrence of HF. However, in practice, the increased mitophagy results in the removal of superabundant mitochondria, leading to inadequate supply of energy for cardiomyocytes with residual mitochondria that is detrimental for patients with HF. As a mediator of mitophagy, BNIP3 is activated under hypoxic conditions and elevated in an *in vitro* model of chronic HF [85]. DRP1 involves mitochondrial fission, and the Parkin recruitment composes the protective response of mitophagy [86]. Once DRP1 is disrupted, mitochondrial elongation is accompanied by mitophagy inhibition, which further leads to cardiac dysfunction and elevated sensitivity to I/R. Similar phenotypes are observed in the case of the tamoxifen-inducible cardiac-specific DRP1-KO mouse model [76].

The vital role of mitophagy in HF progression is also indicated by patients with HF and AMPK *α*2 genetically modified mouse models [87]. The Parkin phosphorylation at serine 495 is essential for the translocation of Parkin to the mitochondria to trigger the occurrence of mitophagy in HF conditions [88]. In addition, the protein level of PINK1 is found to be markedly inhibited in end-stage HF, suggesting inadequate mitophagy [89]. As demonstrated by Hoshino et al., mitophagy impairment promotes HF progression in mice through the recruitment of p53 to Parkin and p53 sequestration [90]. This process leads to damage to the recycling of dysfunctional mitochondria and progression of cardiac dysfunction. Recent evidence also focuses on the role of ULK1-dependent mitophagy against HF induced by pressure overload [57].

Most previous studies believed mitophagy as a cardioprotective process because of the effective removal of dysfunctional mitochondria. However, paradoxically, others believe that the mitophagy pathway is detrimental in the process of HF because mitophagy activation induces the initiation of fibrosis in HF instead of compensation in cardiac hypertrophy.

### 7.5. Arrhythmia

As a prominent component of heart disease, arrhythmia may occur alone or in association with other cardiac complications. Coordinate electrical transmission contributes to normal heart function. Once the cardiac electrical transmission fails, cardiac arrhythmia occurs. To date, the role of mitophagy in arrhythmia is unclear although widespread evidence points out that the mitochondrion plays a critical role in arrhythmia due to its ability to produce energy and ROS [91]. The dysfunction of mitochondria affects ATP production and electrical transmission, leading to altered sarcolemmal K^+^ fluxes via ATP-sensitive potassium channels [92]. The reduced production of ROS induces heterogeneity in cardiac action potential. Mitochondrial dysfunction reduces the mitochondrial membrane potential, further lessens ATP, and elevates ROS [93]. Subsequently, mitochondrial dysfunction is worsened concomitant with reduced mitochondrial membrane potential and ATP production. This futile cycle contributes to electrophysiological alterations and ultimately causes arrhythmia. Therefore, appropriate mitophagy is essential for the homeostasis of mitochondria and elimination of poisonous substances. The alteration of ATP and ROS levels in arrhythmia induced by mitochondrial damage plays an important part in mitophagy [94]. Thus, mitophagy may inhibit or slow down the progression of arrhythmia by removing damaged mitochondria and modulating ATP and ROS levels (Table 1).

## 8. Therapeutic Application of Mitophagy in Heart Diseases

**Figure 3:**
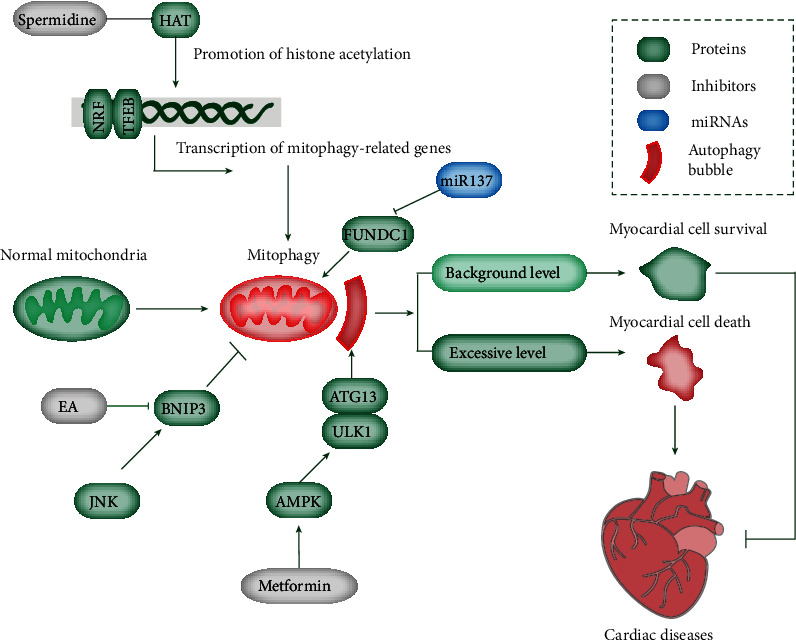
Overview of different mitophagy-modulating compounds and their targets. Mitophagy is regulated by multiple factors. For example, spermidine exerts cardioprotection through inhibited acetyltransferase EP300 and ATM-driven activation of the PINK1/Parkin-regulated mitophagy pathway. EA treatment and JNK pathway modulate mitophagy through BNIP3 regulation. Metformin prevents cardiomyopathy by activating AMPK and autophagy. miR-137 modulates mitophagy by targeting FUNDC1. The basal level of mitophagy facilitates myocardial cell survival, whereas excessive mitophagy induces cell death, ultimately inducing cardiac diseases.

Given the critical role of mitophagy in the pathogenesis of heart disease, mitophagy acts as a promising therapeutic target (Figure 3). The promotion or inhibition of mitophagy can be used to treat or delay the progression of heart disease, maintain stable accumulation of normal mitochondria, and relieve cell damage. Several inhibitors, such as EA and metformin, target AMPK or JNK pathways, thereby mediating mitophagy. Spermidine targets HAT and affects the transcription of mitophagy-related proteins by altering histone acetylation. miR-137 can target the mitophagy-related protein FUNDC1 in myocardial cells and serve as a target for the treatment of heart diseases (Figure 3).

### 8.1. Clinical Drugs and Chemical Reagents

Mitophagy can be modulated by certain drugs and agents. For instance, melatonin inhibits the opening of mPTP and PINK1/Parkin activation in the endothelial cells of I/R models [95]. Thus, melatonin can protect cell death induced by mitophagy and cardiac microvessels triggered by I/R injury through the inhibition of the mitochondrial fission VDAC1-HK2-mPTP mitophagy axis [95]. The mechanism of melatonin in the mediation of mitophagy restoration in DCM is different and probably due to Parkin translocation and Mst1 inhibition [96]. Consistently, the chronic metformin administration (antidiabetic drugs and a potent autophagy inducer) prevents cardiomyopathy by activating AMPK and autophagy activity in diabetic OVE26 mice [97]. Thus, the activation of AMPK may represent a novel approach for DCM therapy (Figure 3). Moreover, the modulation of heme oxygenase-1 and mitochondrial aldehyde dehydrogenase (ALDH2) serves as activators of AMPK, recovers normal autophagic activity, and protects from cardiomyopathy [98]. Simvastatin promotes the translocation of Parkin and p62/SQSTM1 and activates mitophagy, thus inhibiting the infarct size in cellular and mouse myocardial infarction models [99]. Coenzyme Q exerts its anti-ischemic function by disturbing mitophagy [100]. Liraglutide facilitates myocardial restoration to inhibit myocardial infarction by the upregulation of SIRT1 and Parkin, activation of mitophagy, reduction in cellular oxidative stress, optimum redox state, and mitochondrial homeostasis [101].

Zinc modulates enhanced mitophagy to suppress the production of superoxide mitochondria, decrease mitochondrial membrane potential, and alleviate I/R injury by upregulating PINK1 and Beclin1 [62]. In aged myocardium and animals stimulated with isoproterenol, the restoration of mitophagy with antioxidant TEMPOL pretreatment leads to cardiac recovery through the elevation of PINK1 and Parkin [62]. The sevoflurane downregulation of Parkin makes it a potential therapy for the inhibition of mitophagy, reversion of mitochondrial damage, and further protection of the heart [102]. Curcumin suppresses mitophagy through the inactivation of acetyltransferase p300 and inhibition of histone acetylation and GATA4 transcription levels [103]. Erythorbic acid in combination with anthracycline therapy results in reduced cardiomyocyte mitochondrial damage and necrotic cell death and alleviated oxidative stress and cardiac function through the inhibition of BNIP3-induced mitophagy [62]. Another natural compound, spermidine has shown cardioprotection in mice through mitophagy induction in cardiomyocytes and is associated with the inhibited acetyltransferase EP300 and the ATM-driven activation of the PINK1/Parkin-regulated mitophagy pathway [104] (Figure 3). Additionally, ER stress inducers, like tunicamycin and thapsigargin, may be involved in the protection of ischemic stroke injury through the mediation of mitophagy stimulation [105].

Instead of exerting a beneficial effect on heart disease, mitophagy activation may be detrimental to disease progression. Peroxynitrite treatment aggravates cerebral I/R injury in stroke through the induction of PINK1/Parkin-involved mitophagy and recruitment of Drp1 to injured mitochondria [106]. Therefore, the inhibition of peroxynitrite-mediated mitophagy by naringin, a natural antioxidant, attenuates I/R injury by restraining the translocation of Parkin to mitochondria [107].

In addition, several signaling pathways are responsible for the regulation of molecules involved in mitophagy. Rab5-positive early endosomes by the ESCRT- and Parkin-dependent mechanism are responsible for the sequestration of damaged mitochondria in cardiomyocytes [108]. The ablation of Rab5 results in the increased sensitivity of embryonic fibroblasts and cardiomyocytes to cell death. The p53-TIGAR axis is involved in the inhibition of mitophagy by the downregulation of BNIP3, further promoting the accumulation of damaged mitochondria and attenuated cardioprotective effect [109]. Also, BNIP3 can be modulated by the JNK signaling pathway in HF models. As a regulator of the FOXO3a transcription factor, JNK regulates mitophagy through the modulation of BNIP3 levels [110].

### 8.2. Antagonists/Agonists and Gene Knockin/Knockout

Mitophagy can also be modulated by the antagonist of certain receptors, inhibitors of upstream molecules, and mitophagy-related genes. STAT1 acts as a binding molecule of LC3b at the mitochondria. Thus, STAT1 reduces mitophagy and accelerates cell death during myocardial I/R stimulation [111]. The agonist of G protein-coupled estrogen receptor 1 (GPER) promotes mitophagy inhibition, the integrity of mitochondria structure and function, and heart protection against I/R injury through the activation of GPER, decreased translocation of Parkin to the mitochondria, reduced PINK1 protein level, and inhibition of the PINK1/Parkin pathway [27]. The activation of ALDH2 inhibits phosphatase and PINK1/Parkin expression and 4-hydroxynonenal, ROS, and mitochondrial superoxide accumulation and modulates autophagy to defend the heart from I/R injury in I/R rats and hypoxia/reoxygenation H9C2 cells [112]. The Sirt3 overexpression is essential in the occurrence and progression of DCM via the deacetylation activity on FOXO3a and activation of Parkin expression and Parkin-related mitophagy [113]. The Mst1 ablation dramatically facilitates the elevation of Parkin, mitochondrial translocation, and the protection of the myocardium in DCM, which is associated with Sirt3 downregulation [114]. BAG3 depletion results in reduced PINK1/Parkin-dependent mitophagy and aberrant removal of defective mitochondria, contributing to the increased accumulation of toxic substances and cell death under HF by the upstream modulation of Parkin [7]. Given that the ablation of CsA and PINK1 notably reduces PINK1 and Parkin protein levels, CsA and PINK1 can be used to inhibit mitophagy and cell senescence in cardiomyocytes. The Akt2 knockout protects against cardiac aging through the upregulation of Foxo1-mediated BNIP3, PINK1, and Parkin and maintains mitochondrial integrity [62]. The depletion of NIX leads to damaged mitophagy and exacerbated ischemic stroke, whereas the NIX overexpression reverses these phenotypes [115]. The DUSP1 overexpression protects the myocardial tissue injury after I/R by inhibiting BNIP3 activation, deactivating the JNK pathway, and repressing mitophagy [116]. The FUNDC1 ablation in I/R mice induces inhibited mitophagy and aggravated cardiac injury [117].

### 8.3. MicroRNAs (miRNAs)

As small, single-stranded noncoding RNAs, miRNAs are principally involved in the translation inhibition and degradation of targeted mRNAs. A multitude of miRNAs are aberrantly expressed in cardiac diseases. For example, miR-410 is evidently elevated in the I/R injury model in human adult cardiomyocytes, which is accompanied by weakening mitochondrial function and mitophagy [118]. The overexpression of miR-410 exhibits reduced cell viability, ATP generation, mitochondrial membrane potential, and mitophagy. By contrast, the downregulation of miR-410 shows opposite features. HSPB1 is identified to be the direct target of miR-410 and modulates autophagy [114]. As a modulator of NIX and FUNDC1, miR-137 is upregulated upon hypoxia and destroys mitophagy [119] (Figure 3). miR-133a is low in diabetic mouse models and is accompanied by elevated NIX. The overexpression of miR-133a suppresses the translation of NIX and stabilizes the mitochondrial membrane potential [62].

### 8.4. Long Noncoding RNAs (lncRNAs)

Long noncoding RNAs (lncRNAs) are a type of RNA with transcripts exceeding 200 nucleotides that are not translated into proteins. Their roles in mitophagy have been studied. For example, lncRNA H19 governed mitophagy and restored mitochondrial respiration in the heart through the Pink1/Parkin pathway during obesity [120]. Also, the lncRNA Malat1 regulated microvascular function after myocardial infarction in mice via miR-26b-5p/Mfn1 axis-mediated mitochondrial dynamics. Depletion of Malat1 further increased mitophagy, as evidenced by more enhanced expression of Parkin and PINK1 [121].

### 8.5. Circular RNA (circRNA)

Changes in the expression of circRNAs have been reported in a number of diseases, including cancer, heart disease, and neurological disorders. The specific functions of cirRNA are also identified in the mitophagy process. The circRNA ZNF292 alleviated oxygen glucose deprivation-induced injury in rat cardiomyocytes via targeting BNIP3, and BNIP3 further promoted the production of ROS by activating mitophagy in the ischemic myocardium [122]. MFN2 is the host gene of hsa_circRNA_100053. MFN2 could regulate heart failure-related mitophagy by altering the mitochondrial membrane potential, suggesting the role of circRNA_100053 in mitophagy [123]. In addition, the circRNA ACR attenuated myocardial ischemia/reperfusion injury by suppressing autophagy via modulation of the Pink1/FAM65B pathway [124].

### 8.6. Environmental Stimuli

Mitophagy can also be modulated by some specific environmental stimuli. FUNDC1-mediated mitophagy can be induced by hypoxia, which can alleviate I/R preconditioned cardiac injury [125]. BNIP3-related mitophagy is remarkably upregulated in response to acute exercise, which makes mitochondria adapt to this stress and alleviates myocardial injury [126]. The upregulation of Parkin-mediated mitophagy induced by exercise preconditioning inhibits hypoxic-ischemic injury induced by excessive exercise [127].

## 9. Conclusion and Outlook

The abnormal function and damage of mitochondria remain the main pathogenesis of heart disease. Under the action of external stimuli, the mitochondria in cardiomyocytes are depolarized and damaged. The damaged mitochondria are specifically encapsulated in autophagosomes and fused with lysosomes to complete degradation, thereby playing an important role in maintaining the normal function and morphology of cells. At present, mitochondrial autophagy is a hot topic among the molecular mechanisms of organelle-specific autophagy. The induction of mitophagy can potently alleviate damaged mitochondria and maintain adequate normal mitochondria for cell homeostasis. The mechanisms for mitophagy are complicated, interlinked, and tightly regulated by multiple factors. These factors function during essential biological processes, including cell growth, development, aging, and cell death. The aberrant regulation of mitophagy may affect normal cellular function and influence heart disease, thereby making mitophagy a promising therapeutic target for heart disease.

However, whether the induction or inhibition of mitophagy is beneficial for the pathogenesis of heart disease remains to be seen. Moreover, whether mitophagy is contributed by the distinct features of different molecules remains unclear. Future investigations are essential for the validation of mutual effects of various molecules involved in mitophagy induction and the function of mitophagy in heart disease, ultimately making progress in the development of therapeutics in heart disease. Although no magic bullet is currently available to improve mitochondrial quality control, further insight into the molecular mechanisms of mitochondrial autophagy can help in understanding how to counteract the inhibition of mitochondrial autophagy and ultimately prevent these mutations from causing disease.

## Figures and Tables

**Table 1:** Summary of mitophagy pathways in cardiac diseases.

Pathology	Mechanism of action
Ischemic heart disease	The ablation of PINK1 in mice induced cardiac hypertrophy at 2 months of age, and PINK1 knockout aggravated infarct size after I/R injury
	During I/R injury, BNIP3 is upregulated and FUNDC1 is downregulated
	Fundc1-knockout (KO) platelets present impaired mitochondria, which cause more I/R heart injury
	The depletion of Pgam5 in mice showed augmented infarct volume, well correlated with mitophagy inhibition
Diabetic cardiomyopathy	Mitochondrial ROS production was elevated, and BNIP3 level was inhibited in prediabetic rats
	PINK1 and Parkin levels were suppressed in both type 1 and type 2 diabetes models. Parkin ablation mice displayed enhanced mitophagy and serious cardiac hypertrophy under high-fat diet stress
	TAT-Beclin1 contributed to the suppression of cardiomyopathy development
Cardiac hypertrophy	Parkin-depleted mice exhibited elevated cardiac remodeling, and PINK1-ablated mice showed abnormal mitochondrial function, increased oxidative stress, and pathological hypertrophy
	The depletion of BNIP3 also exhibited cardiac hypertrophy at old age
Heart failure	Beclin1 and LC3-II were reduced in the specimen of HF patients
	MFN2 is reported to modulate HF-associated mitophagy via changing the mitochondrial membrane potential
	PINK1 downregulation is commonly observed in HF patients. PINK1-depleted mice were more liable to heart stress and heart failure induced by overload
	NIX depletion showed decreased myocardial fibrosis and more normal systolic function in stress-induced HF mice models
	BNIP3 is activated under hypoxia condition and elevated in an in vitro model of chronic HF
Arrhythmia	Decrease in mitophagy leads to proarrhythmic spontaneous Ca^2+^ release via oxidized RyR2s by mito-ROS

## Data Availability

All data generated or analyzed in this study are available from the corresponding author on reasonable request.
